# Missed meal boluses and poorer glycemic control impact on neurocognitive function may be associated with white matter integrity in adolescents with type 1 diabetes

**DOI:** 10.3389/fendo.2023.1141085

**Published:** 2023-04-05

**Authors:** Edna Litmanovitch, Ronny Geva, Avital Leshem, Mirit Lezinger, Eli Heyman, Maor Gidron, Jessica Yarmolovsky, Efrat Sasson, Sigal Tal, Marianna Rachmiel

**Affiliations:** ^1^ The Gonda Multidisciplinary Brain Research Center, Bar Ilan University, Ramat Gan, Israel; ^2^ Department of Psychology, The Developmental Neuropsychology Lab, Bar Ilan University, Ramat Gan, Israel; ^3^ Pediatric Endocrinology and Diabetes Institute, Shamir (Assaf Harofeh) Medical Center, Be'er Ya'akov, Israel; ^4^ Pediatric Neurology and Epilepsy Department, Shamir (Assaf Harofeh) Medical Center, Be’er Ya’akov, Israel; ^5^ Sackler School of Medicine, Tel Aviv University, Tel Aviv, Israel; ^6^ Radiology Department, Shamir (Assaf Harofeh) Medical Center, Be'er Ya'akov, Israel

**Keywords:** type 1 diabetes, glycemic control, adolescents, brain domains, diffusion tension imaging, cognitive performance

## Abstract

**Background:**

The notion that pediatric type 1 diabetes impacts brain function and structure early in life is of great concern. Neurological manifestations, including neurocognitive and behavioral symptoms, may be present from childhood, initially mild and undetectable in daily life. Despite intensive management and technological therapeutic interventions, most pediatric patients do not achieve glycemic control targets for HbA1c. One of the most common causes of such poor control and frequent transient hyperglycemic episodes may be lifestyle factors, including missed meal boluses.

**Objective:**

The aim of this study was to assess the association between specific neurocognitive accomplishments—learning and memory, inhibition ability learning, and verbal and semantic memory—during meals with and without bolusing, correlated to diffusion tensor imaging measurements of major related tracts, and glycemic control in adolescents with type 1 diabetes compared with their healthy siblings of similar age.

**Study design and methods:**

This is a case–control study of 12- to 18-year-old patients with type 1 diabetes (*N* = 17, 8 male patients, diabetes duration of 6.53 ± 4.1 years) and their healthy siblings (*N* = 13). All were hospitalized for 30 h for continuous glucose monitoring and repeated neurocognitive tests as a function of a missed or appropriate pre-meal bolus. This situation was mimicked by controlled, patient blinded manipulation of lunch pre-meal bolus administration to enable capillary glucose level of <180 mg/dl and to >240 mg/d 2 hours after similar meals, at a similar time. The diabetes team randomly and blindly manipulated post-lunch glucose levels by subcutaneous injection of either rapid-acting insulin or 0.9% NaCl solution before lunch. A specific neurocognitive test battery was performed twice, after each manipulation, and its results were compared, along with additional neurocognitive tasks administered during hospitalization without insulin manipulation. Participants underwent brain imaging, including diffusion tensor imaging and tractography.

**Results:**

A significant association was demonstrated between glycemic control and performance in the domains of executive functions, inhibition ability, learning and verbal memory, and semantic memory. Inhibition ability was specifically related to food management. Poorer glycemic control (>8.3%) was associated with a slower reaction time.

**Conclusion:**

These findings highlight the potential impairment of brain networks responsible for learning, memory, and controlled reactivity to food in adolescents with type 1 diabetes whose glycemic control is poor.

## Introduction

The notion that diabetes mellitus (DM), one of the most prevalent chronic conditions in youngsters, impacts brain function and structure is far from new (1, 2). The theory was first proposed in 1922 (3) and has intrigued many investigators since then, especially regarding its effect on the quality of life. Type 1 diabetes (T1D) remains incurable, the outcome of an autoimmune assault on the insulin-producing pancreatic beta cells in genetically susceptible children (4, 5). Despite intensive management and technological interventions in therapy, most pediatric T1D patients fail to achieve glycemic control goals (6, 7), mainly due to inaccurate, late, or a lack of meal boluses. This may lead to a poorer prognosis for long-term diabetic complications (8). Neurological manifestations, including neurocognitive and behavioral complications, may appear soon after disease onset during childhood and adolescence (9). Studies show that brain volume alterations are detectable in childhood and have long-term influences on adulthood (10, 11).

Although the association between T1D and neurocognitive impairment is well known, the debate today focuses on which abilities are affected, their onset according to disease acquisition, and the underlying mechanisms. The brains of children and adolescents undergo constant change as they re-modulate into adulthood. In line with these changes, personality and abilities are formed in parallel with the continuously redesigned micro and macro-structure of the brain. It is thus critical to understanding the full impact of T1D on the brain, in addition to the impact of glycemic control and, in particular, of glycemic excursions (12), especially in children and adolescents. Glucose is the main brain fuel. Its uptake in the brains of young children reaches adult levels at the age of 2 and is almost double that by 5 years of age, falling back to adult levels at approximately 10 years of age (13). Approximately 25% of total adult glucose consumption is used for brain metabolism (14). These figures suggest that the brain may be vulnerable to glycemic extremes, especially during the first two decades of development (13). Missed meal boluses are known to be both frequent and devastating in the long term in adolescents with T1D and poor glycemic control. Furthermore, a significant association has been demonstrated between frequently missed boluses in pediatric T1D patients with poor glycemic control, and complications (8, 15).

A study that combines the parameters of acute glucose excursions and chronic glycemic control with neurocognitive assessment is thus required. As reported in the review we performed when preparing the study protocol (1), more than 40 years of diabetes research have demonstrated brain alterations and an increased risk of cognitive decline in T1D (16). These findings can now be corroborated by exploring changes in neural network activity using methods such as diffusion tensor imaging (DTI). Conventional MR techniques, such as T1- and T2-based measurements, cannot provide detailed information about the integrity and location of white matter (WM) tracts. DTI provides unique biologically and clinically relevant information for the study of diabetes-related alterations in the integrity of neuronal pathways (17–24). Based on MR measurement of the speed of water diffusion in tissues, it enables the characterization of tissue composition, physical properties, and architectural organization (25). With tractography, WM pathways can be traced *in vivo*, permitting the study of the nature of damage to WM tracts.

The overarching hypothesis of the association between acute transient and long-term hyperglycemia, explored in the research described here, is an anticipated relationship between neurocognitive performance in adolescent patients with T1D, as influenced by diabetes glycemic control, and its association with the quantitative parameters of WM in specific major pathways.

## Materials and methods

### Study design

This report includes findings from a wider, case-control proof-of-concept study conducted at the Pediatric Neurology and Epilepsy Department Research Unit (PNRU) at the Shamir (Assaf Harofeh) Medical Centre (SHMC), Be'er Ya'akov, in collaboration with SHMC’s Neuroradiology Institute and Pediatric Endocrinology and Diabetes Institute, the Hadassah Medical Center’s Neurology Department, and Bar Ilan University’s Gonda Multidisciplinary Brain Research Center. The study observed the Helsinki Declaration’s ethical principles for biomedical research involving human patients, together with local and national regulations. Prior to enrollment, all participating institutions obtained approval from their ethics committees and Israel’s Health Ministry’s Helsinki Committee. The study, with its full protocol, is listed at www.clinicaltrials.gov (NCT02923323).

### Study population

The study population comprised 12- to 18-year-old T1D patients who were being cared for at SHMC’s Pediatric Endocrinology and Diabetes Mellitus Institute, along with their healthy siblings of similar age. Healthy siblings, sharing close genetic profiles and similar environments, were a natural control group. Inclusion criteria for the T1D group were a T1D diagnosis according to ADA criteria (26) and a basal-bolus regimen for more than 2 years. Exclusion criteria were more than one severe hypoglycemic event or more than one episode of diabetic ketoacidosis (DKA), other than at diagnosis. The exclusion criteria for all participants comprised a history of head injury, epileptic episodes, psychiatric medications, renal or liver function abnormalities, and language limitation. The study population was divided into three groups: healthy control siblings, T1D patients with good glycemic control, and T1D patients with poor glycemic control. Glycemic control clusters were defined as glycated hemoglobin (HbA1c) above 8.3% as poor control, and HbA1c ≤ 8.3% as good control according to the EXCHANGE study results, with a mean teenager HbA1c of 8.26% in a large population (27).

Out of the 31 adolescents recruited, 8 were in the better glycemic control group, 9 were in the poorer glycemic control group, and 13 healthy siblings comprised the control group. One T1D participant from the better-controlled group was excluded due to incidental abnormal MRI scans. Five participants (two with T1D and three controls) did not undergo MRI for technical reasons.

### Study setup: Three sessions performed in 1–4 weeks

Session 1 comprised signing informed consent by parents and participants and obtaining medical histories, physical assessments, baseline cognitive and lingual readiness by parents and participants, and cognitive assessments of participants.

Session 2 was a 30-h stay at the PNRU for neurocognitive assessments while monitoring food intake, glucose levels, and insulin administration. A specific neurocognitive test battery was performed twice, each time 2 h after lunch—at glucose >240 mg/dl and glucose ≤180 mg/dl. The glucose level was randomly and blindly manipulated before lunchtime tasks: rapid-acting insulin was administered before lunch on one day, and an injection of 0.9% NaCl solution was given before a lunch bolus on another day. Additional tasks were performed without insulin manipulation during hospitalization.

Session 3 comprised brain imaging with a prior capillary glucose measurement to verify levels of 70–240 mg/dl.

### Measurements

Clinical data were retrieved from medical files; they included demographic information :age, gender, and socioeconomic status by home address. The SEP (socioeconomic position) based on home address was analyzed according to the Israel Central Bureau of Statistics Characterization and Classification of Statistical Areas within Municipalities and Local Councils by Socio-Economic Level of the Population, 2015. The SEP index classifies neighborhoods and localities into clusters, with 1 being the lowest rating and 10 being the highest. It is an adjusted calculation of 14 variables that measure social and economic level in four domains—demographics, education, standard of living, and employment. (28), clinical data :diabetes duration, annual HbA1c based on 3 last annual measurements, and complications. Physical examination elicited weight, height, body mass index (BMI), (SDS were calculated by CDC 2000 growth charts) (29), and Tanner staging (30). Glycemic control was defined according to HbA1c (26).

#### Glucose level measurements

ISG was assessed using a blinded CGMS (Minimed Inc., Sylmar, CA). Capillary glucose was measured regularly before meals and 2 hours after, and prior to neurocognitive testing. Patients and the neurocognitive team were blinded to glucose levels measured prior to neurocognitive tests.

#### Neurocognitive and psychosocial measurements

**Figure 1:**
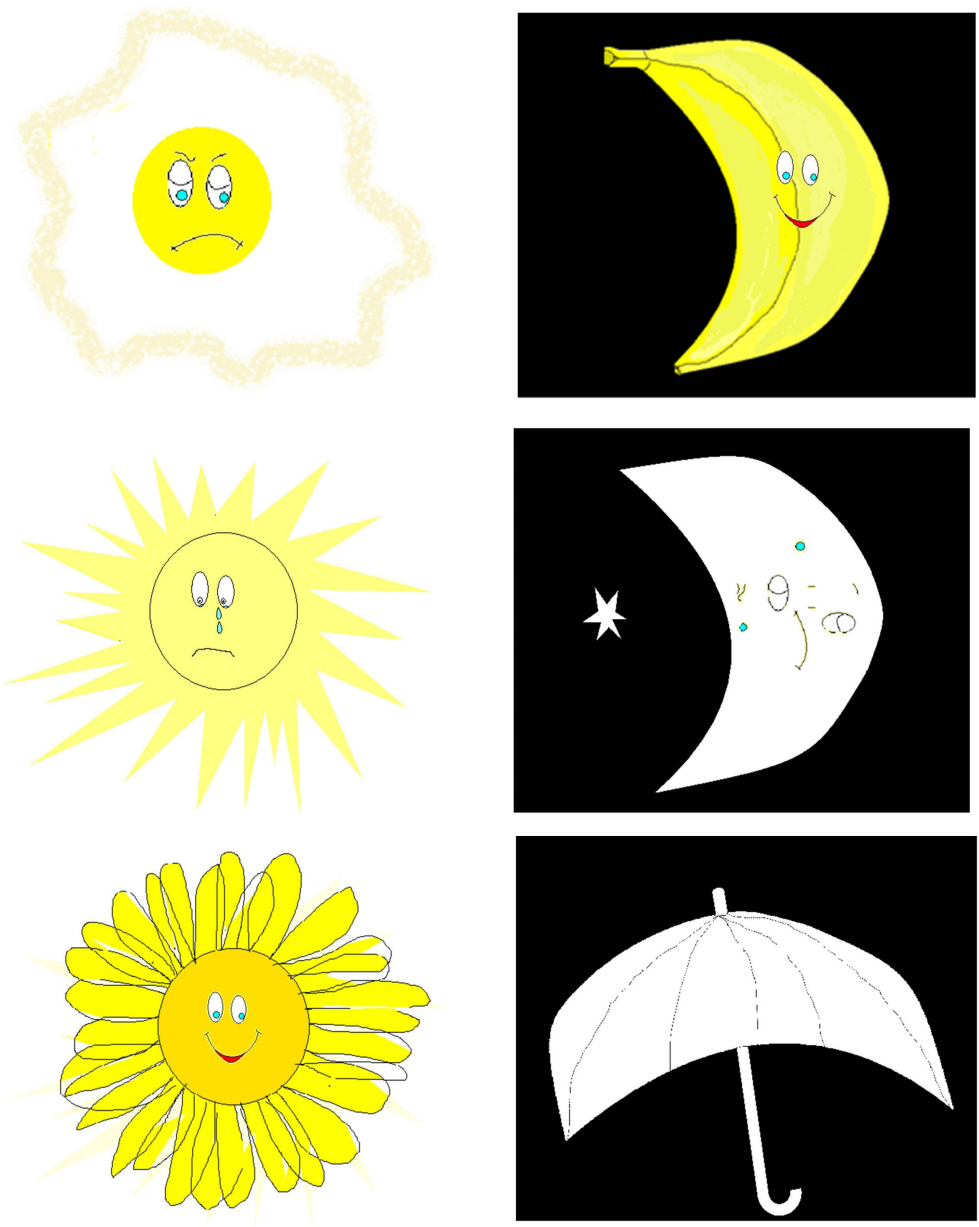
Day–night emotional Stroop stimuli.

**Figure 2:**
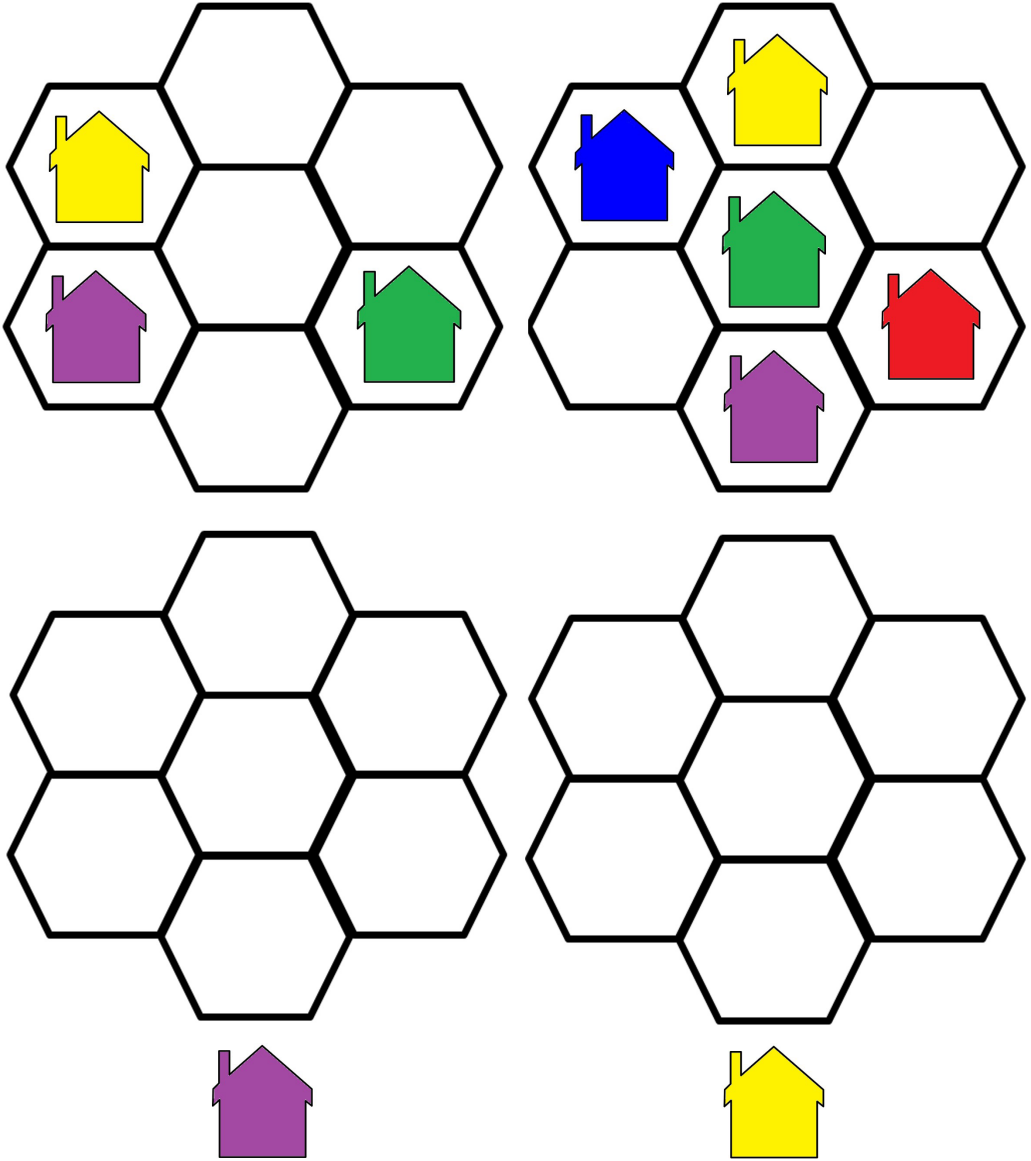
Visual Update Stimulus example.

**Figure 3:**
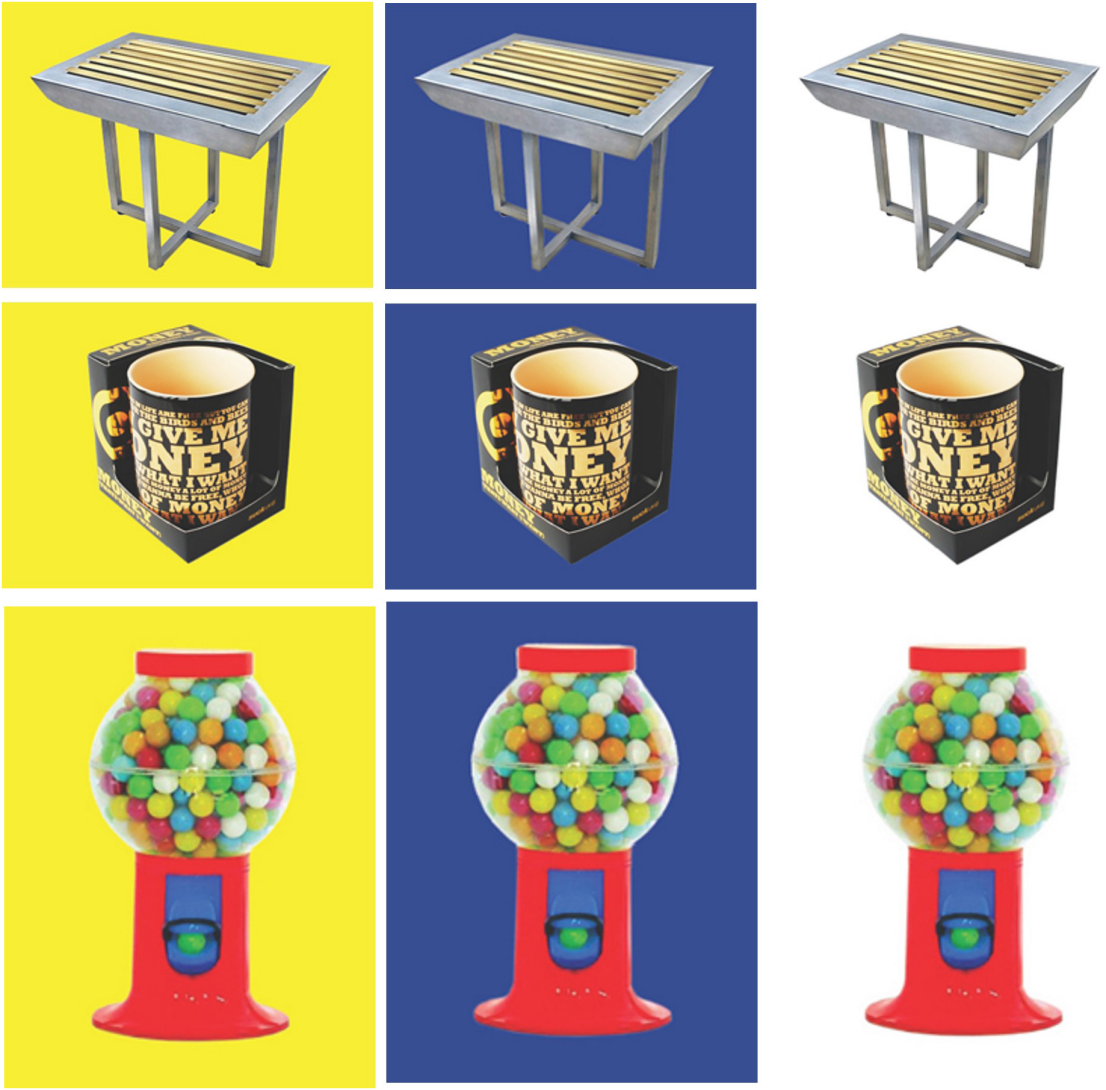
Object Recall examples.

Neurocognitive data included a designed battery of tasks specifically modified for food intake. This report refers to the following tasks:

1. The Word Selective Reminding Test (%) subset 3 of the Test of Memory and Learning (TOMAL-2) (31) measures the ability of learning and immediate verbal recall. The examiner reads off a list of words to the participant, who is encouraged to recall as many of them as possible, regardless of the order of recall. After each trial, the examiner reminds the participant of the forgotten words and reads out the word list again. The subtest ends when the participant remembers every word on two consecutive trials, or after eight trials, regardless of memory proficiency (32).
2. The Day and Night Task—Emotional Stroop for Eating Disorders (EST-ED), which is specifically designed, modified, and computerized to evaluate response inhibition to emotional food-related stimuli. It had two general shapes, each presented in three versions—one signifying an edible item and two as controls. Six stimuli were presented: nonfood pictures (for example, the moon and sun), food pictures (for example, a sweet item, a banana, and a low-sugar item, such as an egg), and two natural pictures (for example, an umbrella and a flower) (**Figure 1**). All items were shown at random with neutral emotion. Participants were instructed to push one button in reaction to stimulations similar to the moon and another in reaction to those similar to the sun (33, 34). The task was specifically modified for the study. Dependent measures analyzed for the study’s purposes were accuracy and response time in emotional Stroop response (sun–moon), emotional nonfood Stroop (umbrella–flower), and emotional food Stroop (banana–egg).
3. The Visual Update Task is a spatial–figural updating task that evaluates the executive function of updating and the monitoring of working memory representations often associated with the prefrontal cortex dorsolateral section. To succeed, participants must monitor and code relevant incoming information and correctly adjust items held in their working memory by replacing old information with that which is newer and more pertinent (35). Our modified computerized task consisted of differently colored house shapes presented in different positions within a flower-shaped frame. Depending on the trial’s load level, two to five different colors were used. The colored houses were presented one at a time, with participants asked to keep track of the last position of each color. At recall, the differently colored houses that had been shown were presented again, one at a time within the frame. Participants responded by clicking the mouse in the area of the frame where the color had appeared (**Figure 2**).
4. The Object Recall Task is a computerized semantic memory task in which new objects with different casings are presented on two possible backgrounds: blue (calming arousal) and yellow (exciting arousal). The task, described in an unpublished thesis by Tamar Schwarz of Bar Ilan’s Department of Psychology, is based on an fMRI paradigm of semantic object representation (36) that was modified for our study. Initially, a series of objects shown against a colored background were presented to the participant, who was asked to remember as many as possible (**Figure 3**). The objects were then presented again, in succession, without backgrounds, and the participant was required to indicate whether they had been previously presented and whether against a yellow or blue background. Semantic memory of visually presented recall objects involves the thalamus, pre-supplementary motor area, and several other somatic cortical regions (37). We analyzed responses for accuracy and response time.

Data were anonymized and coded by the neurocognitive team prior to analysis, according to clinical and glycemic measures.

#### Brain imaging measurements

Magnetic resonance imaging (MRI) scans were performed on a Siemens Medical Solutions 3.0-Tesla MRI scanner at SHMC. The T1 scans were used as an anatomical reference, while DTI was used to map neuronal tracts and evaluate brain WM properties. This was performed without sedation or contrast material.

#### MR data acquisition

T1 data were collected at a spatial resolution of 0.8 × 0.8 mm voxels, with 0.9-mm-thick axial slices covering the entire brain with no gaps. Repetition Time/Echo Time (TR/TE) = 2,000/2.41 ms, field of view (FOV) 137 = 245 mm, matrix size = 287 × 287. Scanning time was approximately 5 min.

DTI data acquisition protocol was as follows: *b*-value = 1,000 s/mm^2^, along 30 non-collinear gradient directions (plus one b0 image), TR/TE = 15,000/91 ms, matrix size = 113 × 113, and a flip angle of 90°. Spatial resolution was 1.5 × 1.5 mm voxels, with 1.5-mm-thick axial slices with no gaps covering the entire cortex. Scanning time was about 9 min. Data were analyzed using MATLAB and C++-based software tools—SPM software (version 12, UCL, Queen Square Institute of Neurology, London, UK) and mrVista packages (http://white.stanford.edu/newlm/index.php/Software). This included correction for head movement and image artifacts, and the normalization and creation of a reference volume using a T1-weighted, AC-PC-aligned image. T1-weighted images were used for grey matter (GM) and WM volume assessment.

#### DTI data preprocessing

Using the mrDiffusion package from VISTA, the DTI data preprocessing pipeline had three additional steps:

1. Correct DTI data for eddy current and movement noise and align these to the anatomical reference.
2. For each voxel in the scanned volume, fit a tensor model based on a Gaussian diffusion signal decay model and linear least-squares fits. Then, extract the three eigenvalues (Λ_1_, Λ_2_, and Λ_3_) by tensor diagonalization and calculate the FA (an index that reflects the orientation of diffusion—mainly the uniform directionality of the tract, and it is high along well-defined pathways), AD (the rate of diffusion in the principal diffusion direction of the voxel), and RD (the rate of diffusion perpendicular to the principal diffusion direction of the voxel) according to the following equations:

(1) $$\text{Fractional anisotropy}=\sqrt{\frac{3}{2}}\frac{\sqrt{{\left(\lambda 1-\bar{\lambda }\right)}^{2}+{\left(\lambda 2-\bar{\lambda }\right)}^{2}+{\left(\lambda 3-\bar{\lambda }\right)}^{2}}}{\sqrt{\lambda 1^{2}+\lambda 2^{2}+\lambda 3^{2}}}$$

(2) $$\text{Axial diffusivity}=\lambda_{1}$$

(3) $$\text{Radial diffusivity}=(\lambda_{2}+\lambda_{3})/2$$

3. From the tensors created in the preprocessing procedure, create DTI maps (FA, RD, and AD) for each participant.

#### Fiber tracking and quantification

Automated fiber quantification (AFQ) (open source and freely available at https://github.com/yeatmanlab/AFQ) was used for the automated identification and quantification of cerebral WM pathways. This software package uses mrDiffusion functions and specially built functions and scripts executed with MATLAB. As first published in 2012, the AFQ automated fiber tract segmentation has proven to be equivalent to the time-consuming manual techniques that served as the gold standard (38). It is now widely used in clinical trials but has a particular advantage in this study as it was demonstrated in healthy children and adolescents.

**Figure 4:**
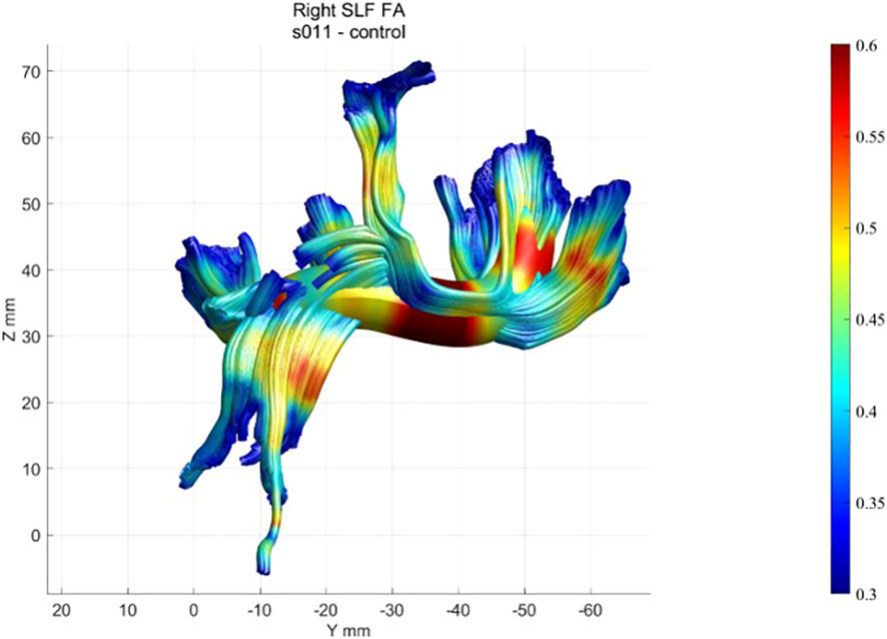
Example of a healthy participant’s FA profile of the right SLF.

First, tracing is initialized from the hemisphere mask: eight seed points are placed at equidistant locations in all voxels with an FA value greater than 0.3. Fiber tracts are estimated using a deterministic streamlined tracking algorithm (39) with a fourth-order Runge–Kutta path integration method. For tracking purposes, a continuous tensor field is estimated using trilinear interpolation of the tensor elements. Paths were tracked with a 1-mm step size; the stopping criterion was FA< 0.2 or tracking angle > 30°. The methodology and algorithms for the automated segmentation, tract cleaning, and tract quantification procedures are described elsewhere (38). AFQ was used to trace 20 tracts, including corpus callosum segments, corticospinal tract, thalamic radiation, cingulum cingulate, cingulum hippocampus, superior longitudinal fasciculus, inferior longitudinal fasciculus, inferior fronto-occipital fasciculus, uncinate, and the arcuate, and to segment the corpus callosum into eight cortical segments. Each of the 28 tracts was resampled to 100 equally spaced nodes, and diffusion properties were calculated for each node of each fiber (**Figure 4**). The mean and standard deviation were calculated for each diffusion property at each node of each tract for the healthy participants. A confidence interval was generated for each tract to quantify the similarity of each patient to the standard tract profile of the healthy group.

#### Callosal segmentation

The callosal segmentation of regions of interest (ROIs) deep in the WM was based on Huang (40) and Dougherty (41). This protocol was extended in a previous study by dividing the superior frontal segment into two parts, using the medial branching of the precentral sulcus as an anatomical landmark. Based on the cortical destinations of callosal fiber tracts, the corpus callosum was divided into eight segments: orbital, anterior frontal, superior frontal, motor (posterior frontal), superior parietal, posterior parietal, temporal, and occipital. A dual analysis using both ROI-based segmentation and selecting fiber segments was performed with the AFQ software. The original AFQ package was created for the segmentation of 20 major tracts, as defined by Mori et al. (42), with additional WM tracking functions added over the years. Bar Ilan’s Neurolinguistics Lab in the Gonda Multidisciplinary Brain Research Center and Stanford’s Wandell Lab in the Center for Cognitive and Neurobiological Imaging collaborated to add special functions for callosal segmentation to the AFQ software, based on the segmentation described previously. This automated callosal segmentation was validated in the Gonda Neurolinguistics Lab by comparing it with manual segmentation using Quench software (Vista Lab), by visual inspection of the created tracts, and by statistical comparison of the FA extracted using both methods. Callosal segments were clipped 5 mm to each side of the mid-sagittal plane to extract the quantitative properties of the callosal segments of the corpus callosum itself, uninfluenced by distant location. Thirty nodes (representing locations) were defined on every clipped segment to calculate the tract profile. The mean diffusivity parameters for all callosal segments were calculated for each patient with T1D and each healthy participant. FA, AD, and RD were further examined for each segment.

### Outcome measures

The two primary outcome measures were: (a) difference in neurocognitive assessment scores between groups, according to glycemic control, and (b) difference in DTI parameters of AD, RD, and FA and corpus callosum (CC) segments between T1D and healthy groups, according to glycemic control. A secondary outcome measure was the correlation between associations with specific MRI tract alterations and neurocognitive performance scores.

### Statistical analysis

All analyses were performed with MATLAB 2020a (© 1994-2020, The MathWorks, Inc.), with the assistance and guidance of a trained statistician.

Quantitative/numerical measures are presented as means ± SDs and min–max.

Qualitative/categorical measures are presented as percentages.

Participants’ tasks with artifacts or missing data were excluded. After the performance was corrected and normalized, we ensured that no confounding variables, such as age or gender, influenced group differences. Correlations between cognitive performance, neurobehavioral outcome measures, and disease attributes, in addition to analysis of variance (ANOVA), were used to examine differences between T1D (distinguishing between glycemic clusters where relevant), healthy control variables, and neurobehavioral outcome measures. We considered the correlation to be significant when *R* ≥ |0.4| and the *p*-value< 0.05.

Voxel-based analysis (VBA) is a statistical method that detects differences in brain regions on a voxel-by-voxel basis (43) on segmented GM probability maps and DTI maps (FA, RD, AD, and volume). A two-sample *t*-test was used to compare the T1D and healthy groups.

Statistics of AFQ tract profiles: *t*-tests were calculated pointwise along each tract for diffusion properties. Given the high degree of correlation between nearby points, the Bonferroni correction was too conservative (38), and permutation-based multiple comparison correction (44) was used to adjust *p*-values given the structure of the data. This proved significant and resulted in a corrected *p*-value of<0.05.

The correlation between the locations of significant tract group differences and neurocognitive and neurobehavioral task performance was analyzed with MATLAB R2020a. Pearson and Spearman’s correlations were employed using a *p*-value threshold of 0.05. For Pearson correlations, *r* > |0.6| was applied, and for Spearman correlations, *r* > |0.5|.

## Results

The study population comprised 30 adolescents. Of the 17 participants in the T1D group, 8 were boys (mean age, 14.7 ± 1.68 years). Of the 13 participants in the healthy group, 8 were boys (mean age, 14.6 ± 1.73 years). **Table 1** shows their clinical and demographic parameters. Parental and participant baseline neurocognitive and language assessments were within normal ranges, with no significant differences within families.

**Table 1:** Statistics: Clinical and demographic characteristics of the study population.

Group	T1D well-controlled group	T1D poorly controlled group	Healthy group	*p*-value
**Number**	8	9	13	NA
**Gender (boys: girls)**	3:5	5:4	8:5	0.45
**Age** ^†^	14.5 ± 1.91	14.9 ± 1.54	14.64 ± 1.73	0.97
**Puberty** ^†^	3.4 ± 1.51	3.7 ± 1.32	3.54 ± 1.71	0.99
**T1D duration^†^**	5.8 ± 2.83	7.2 ± 5.05	NA	NA
**HbA_1c_%^††^**	7.6 ± 0.59	9.5 ± 1.03	NA	NA
**HbA_1c_ (mmol/mol)**	70.6		NA	NA
**SEP cluster**	6.80 ± 2.11		6.46 ± 1.81	0.66
**SEP index**	0.72 ± 0.92		0.59 ± 0.75	0.70
**Height SDS**	−0.16 ± 0.89		−0.40 ± 1.38	0.56
**BMI SDS**	0.10 ± 0.67		−0.19 ± 0.77	0.27

^†^Mean ± standard deviation.

^††^HbA_1c_ calculated from the last three visits during the past year at session 1.

The SEP (socioeconomic position) based on home address was analyzed according to the Israel Central Bureau of Statistics Characterization and Classification of Statistical Areas within Municipalities and Local Councils by Socio-Economic Level of the Population, 2015. The SEP index classifies neighborhoods and localities into clusters, with 1 being the lowest rating and 10 being the highest. It is an adjusted calculation of 14 variables that measure social and economic level in four domains—demographics, education, standard of living, and employment.

NA, not applicable.

### Neurocognitive performance

One-way ANOVA analysis revealed significant group and cluster differences in cognitive function.

#### Memory and learning

The performance of the TOMAL Word Selective Reminding task differed by group (*p* = 0.03) and by glycemic control (*p* = 0.02) and was associated with ISG (*r* = 0.54, *p* = 0.002). On average, lower HbA1c and lower ISG were associated with better scores.

#### Inhibition ability

Both group differences (*p* = 0.001) and glycemic control differences (*p* = 0.004) were found in the Stroop response time, which was associated with a higher ISG (*r* = 0.65, *p* = 0.003). Stroop non-food and food response times differed between groups (*p* = 0.04 and *p* = 0.02, respectively). Stroop food response time implied a significant difference between glycemic control clusters (*p* = 0.05). The relationship was such that better glycemic control and lower ISG were associated with better inhibition ability. On average, the response time of the T1D group was significantly higher than that of the control group.

#### Executive function performance

We found a significant difference in Visual Update response time by group (*p* = 0.01) and by glycemic control (*p* = 0.04): poorer glycemic control mean response time was 1,463 ms, whereas better glycemic control mean response time was 1,437 ms.

#### Semantic memory

**Figure 5:**
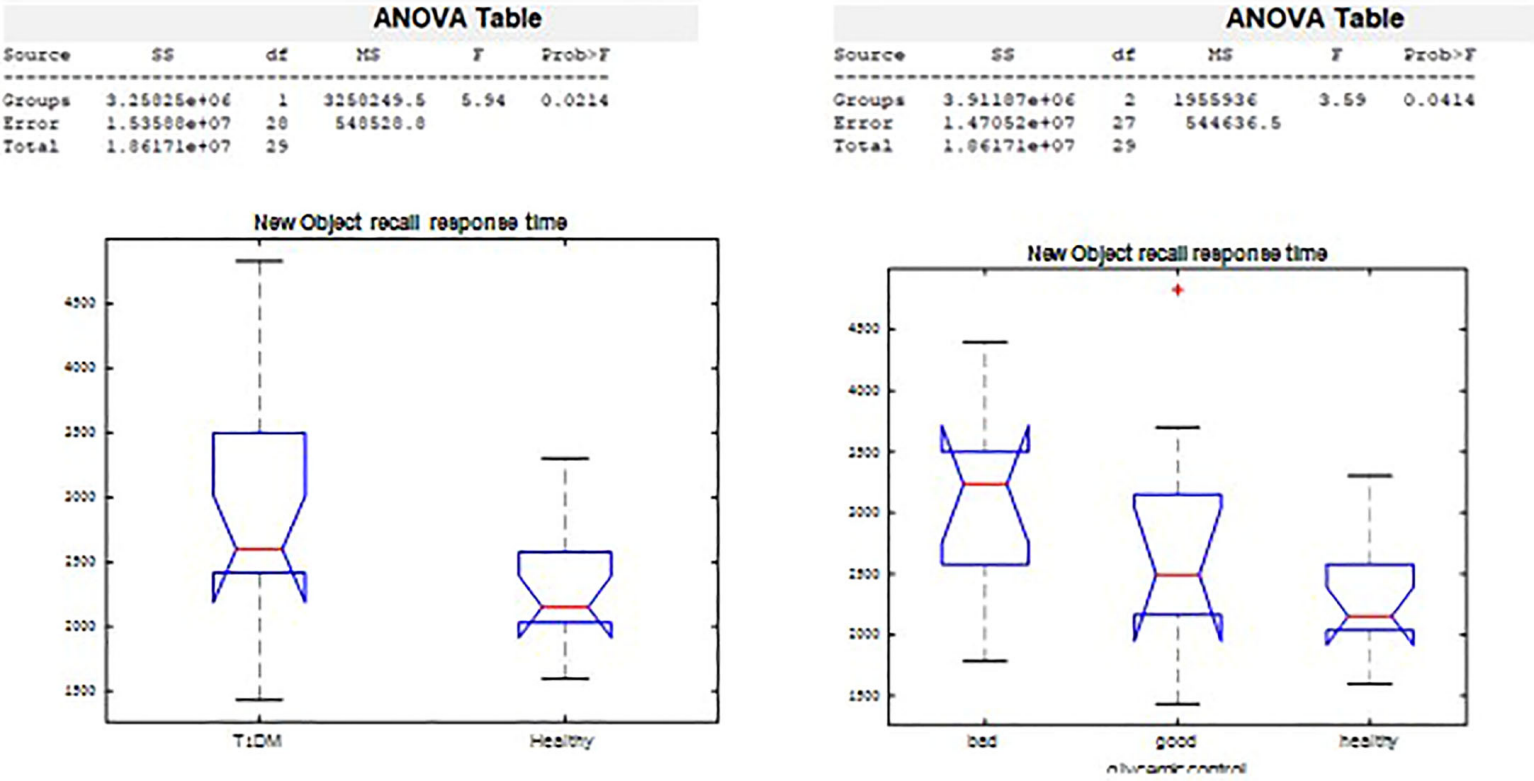
New Object Recall significant differences.

On the New Object Recall Task (**Figure 5**), we observed significant difference in response time by groups (p=0.02), and by glycemic control (p=0.03). The response time in remembering whether the object had been presented was 2,943.2 msec among those with T1D, compared with a response time of 2,278.2 msec in the healthy control group. Poorer glycemic control associated with longer time needed to recall the object.

### Anatomical brain differences between groups

Using VBA, the MRI data of each participant were analyzed and compared between groups. A *t*-test and multiple corrections on all diffusivity measures (FA, AD, and RD) in every tract of the corpus callosum segments and major WM bundles revealed significant differences between groups. FA was significantly lower in the T1D group, mainly in the superior longitudinal fasciculus (SLF) (0.25 ± 0.03 vs. 0.29 ± 0.02. *p* = 0.0001) and corona radiata (CR) (0.32 ± 0.05 vs. 0.38 ± 0.03. *p* = 0.0002). Lower AD was also observed in the T1D group (*p<* 0.005).

**Figure 6:**
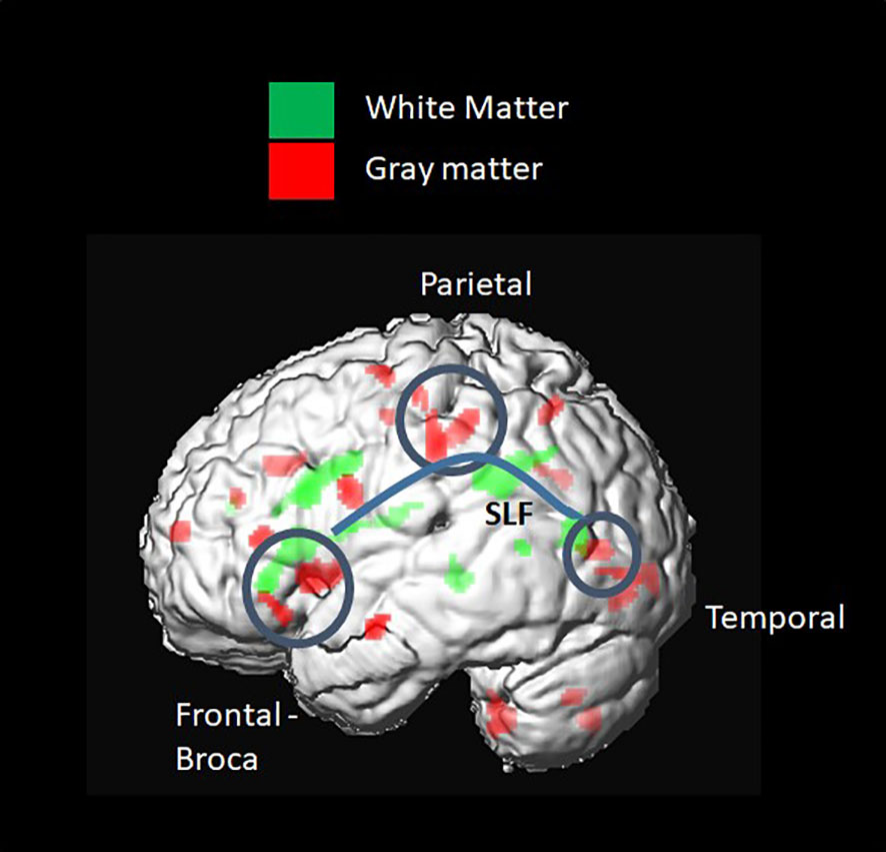
Voxel-based group comparison revealed a higher grey matter (GM) index in the type 1 diabetes (T1D) group. This included brain regions connected by white-matter (WM) fiber tracts, such as the SLF and corona radiata. A higher GM probability index was found in the frontal inferior triangular and operculum regions, known as Wernicke, and in parietal regions, such as the inferior parietal and postcentral gyrus.

GM density measured by the GM probability index was higher in the T1D group in the Broca and Wernicke regions, connected by the WM fiber tracts mentioned above (SLF and CR) (**Figure 6**).

### Comparison of diffusion properties in major brain segments

#### Diffusivity coefficients of corpus callosum clipped segments

**Figure 7:**
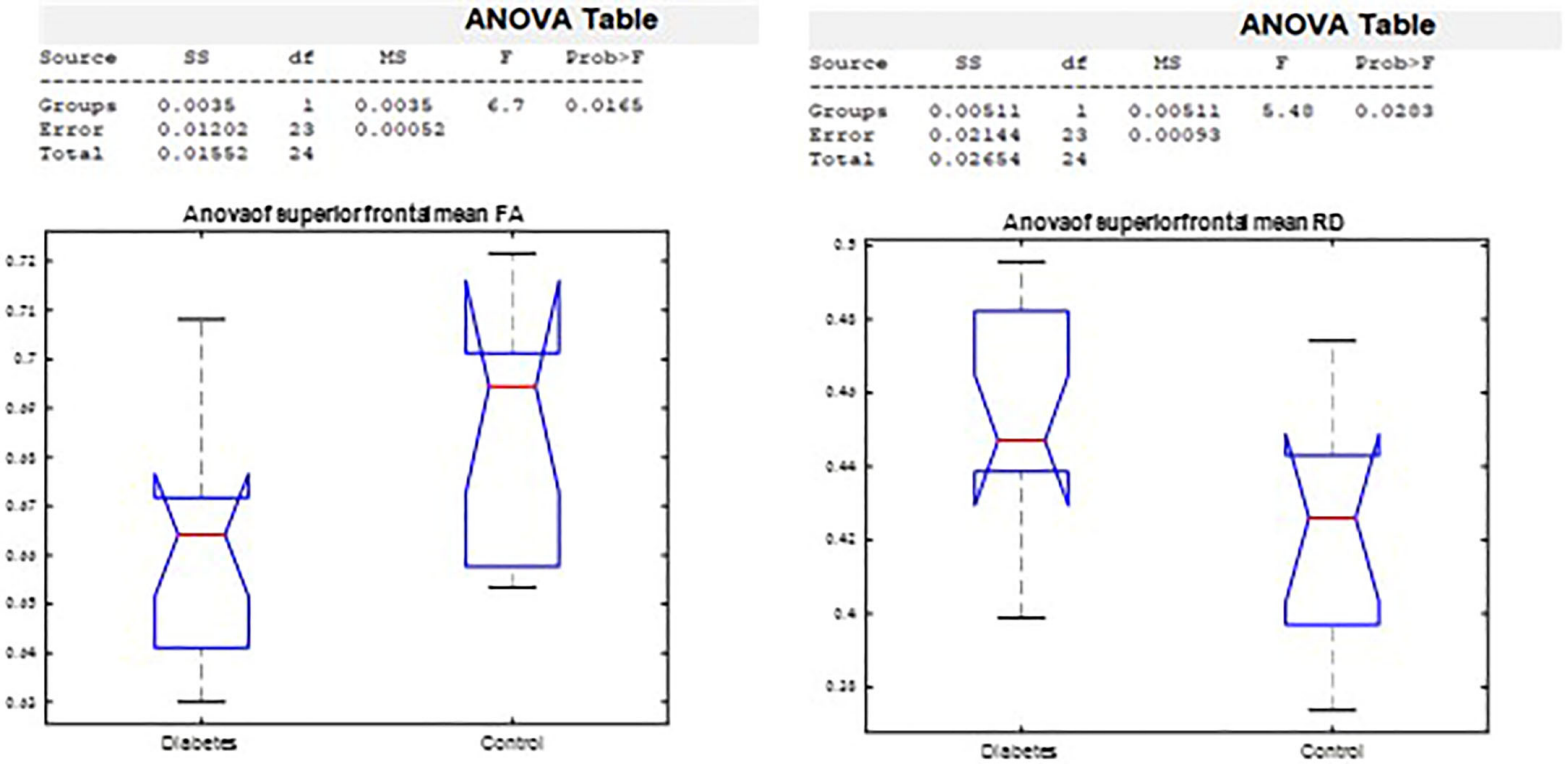
Analysis of variance by group in the superior frontal FA and RD corpus callosum segment center.

The ANOVA on callosal segment diffusion properties revealed a significant difference between the T1D group and the healthy group in the superior frontal callosal segment centers FA (*p* = 0.02) and RD (*p* = 0.03). FA was significantly lower, and RD was significantly higher in the T1D group (**Figure 7**).

#### Diffusivity coefficients of major WM brain tracts

**Figure 8:**
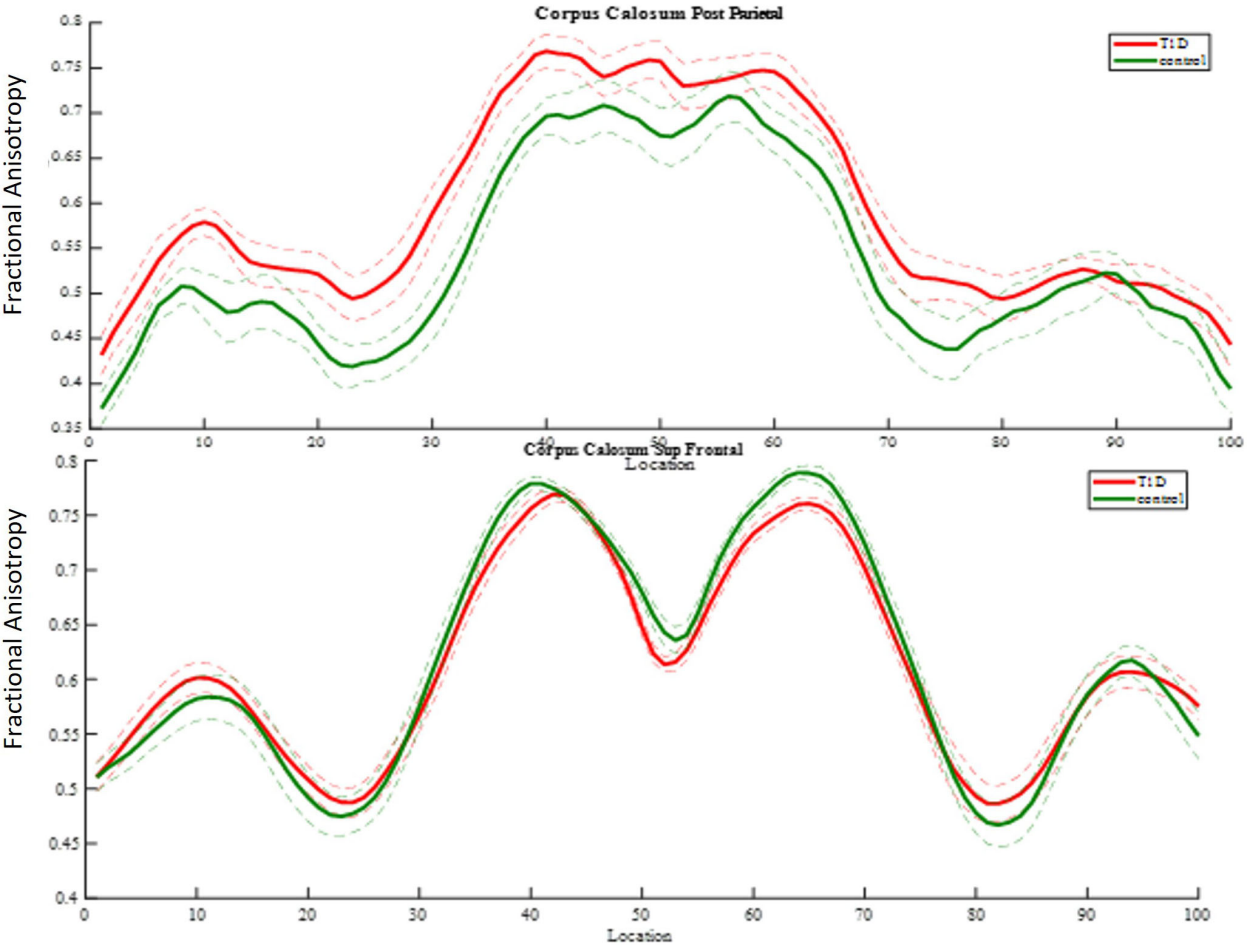
FA group comparison plots. Control group and diabetes mean FA ± 1 SD is plotted along 100 nodes on tract center. The upper graph shows significantly higher FA values in the diabetes group in 32 locations of the posterior parietal callosal segment. The panel beneath it shows significantly lower FA values in the diabetes group in 12 locations of the superior frontal callosal segment.

Using the AFQ framework for quantifying diffusion measurements at multiple locations along the trajectory of major WM tracts, a diffusion measurement “tract profile” was created at anatomically equivalent locations along T1D and healthy brain trajectories. Many locations along the observed tracts revealed group differences and were later associated with neurocognitive performance. A threshold of at least five locations (5%) with significant differences along T1D and healthy trajectories was applied to reveal anatomical group differences and to mark specific bundles. Of the eight fiber groups, the most apparent group differences in mean FA were seen in the superior frontal corpus callosum (*p<* 0.005) and the posterior parietal corpus callosum (*p<* 0.005) (**Figure 8**). Of the nine fiber groups, the most apparent group differences in mean AD were observed in the right superior longitudinal fasciculus (SLF) (*p<* 0.001), left uncinate fasciculus (*p<* 0.04), superior parietal corpus callosum (*p<* 0.005), forceps minor (*p<* 0.005), and anterior frontal corpus callosum (*p<* 0.005). Of the eight fiber groups, the most apparent differences in mean RD were found in the posterior parietal corpus callosum (*p<* 0.001), left arcuate (*p<* 0.003), left cingulum hippocampus (*p<* 0.02) and left SLF (*p<* 0.03). The differences along the tracts were in both directions, but most of the differences were in favor of the control group, suggesting denser and more coherent bundles.

### Correlations between brain diffusion measures and cognitive performance

#### Right SLF quantitative diffusion parameters associated with Stroop response time

Stroop, Stroop food, and Stroop non-food response times correlated with the mean AD of the dorsal part of the right SLF (most significant *r* = −0.77 *p* = 0.005). The relationships were such that lower inhibitory ability was negatively associated with tract coherence.

#### Integrity of the superior frontal segment of the corpus callosum correlated with inhibition ability

Higher mean FA of the superior frontal segment of the corpus callosum, which connects the hemispheres, was significantly negatively correlated (most significant *r* = −i.77, *p* = 0.006) with emotional food Stroop response time. The relationship was such that higher bundle integrity was associated with a better ability to inhibit the emotional response to a food-related stimulus.

#### Posterior parietal corpus callosum density associated with verbal memory

Using Spearman correlation, performance on Word Selective Reminding (%) correlated with the mean RD of the left posterior parietal segment of the corpus callosum connecting the hemispheres (most significant *r* = −0.63 *p* = 0.0008). The relationship was such that higher bundle integrity was associated with better performance.

## Discussion

A significant association was found between glycemic control and performance in the domains of EF, inhibition ability, semantic memory, and learning and verbal memory. Inhibition ability was found to be specifically related to food management. Poorer glycemic control (>8.3%) and greater glycemic excursions following a missed bolus meal were associated with longer reaction times in the cognitive tasks tested and poorer performance in learning and verbal memory. These findings highlight the impairment of brain networks responsible for learning, memory, and controlled food responsiveness in adolescents with T1D.

### Neurobehavioral functions

The hypothesis tested here was that faulty glycemic control in adolescents with T1D, caused by inaccurate, late, or lack of meal boluses (15), may decrease performance even without previous risk factors such as a significant episode of DKA or hypoglycemic seizure (8).

A recent review (16) indicates that cognitive declines in young people with T1D are characterized by overall lower cognitive performance and moderately lower memory, attention, and EF. This is mostly found with early diabetes onset. It took 2 years from diabetes onset to show a moderate decline in these domains, which were still present after 6 and 12 years (45, 46). Collectively, the early age of diabetes onset, higher HbA1c, hypoglycemic events, and DKA around onset were all major contributors to cognitive decline. These factors may cause an initial “strike” to the brain, which later adapts to fluctuating glucose levels (16). Function during blinded CGMS and food-related assignments was not, however, assessed among adolescents.

Our study reveals group differences in inhibition abilities and executive function, with specific differences in the executive control of processing food stimuli. Poorer glycemic control and higher glycemic excursions were associated with poorer cognitive processing, learning, memory, and executive performance, supporting our hypothesized relationship between glycemic control and cognitive performance.

### Brain structure

DTI tractography is considered a sound method for examining delicate differences in brain matter coherence, and we used this to evaluate the association between T1D and brain microstructure properties. The literature contains a variety of methods and study populations, all with inconclusive results concerning T1D and healthy populations regarding GM and WM volume and WM integrity (21, 22, 47). Associations between WM integrity and cognitive performance are sparse. The uniqueness of our study is its in-depth analysis, achieved by using many attributes and large-scale acquired data and performed in genetically and socioeconomically similar populations of adolescents. Several interesting findings and new insights into all these aspects pave the way for future, more focused studies.

**Figure 9:**
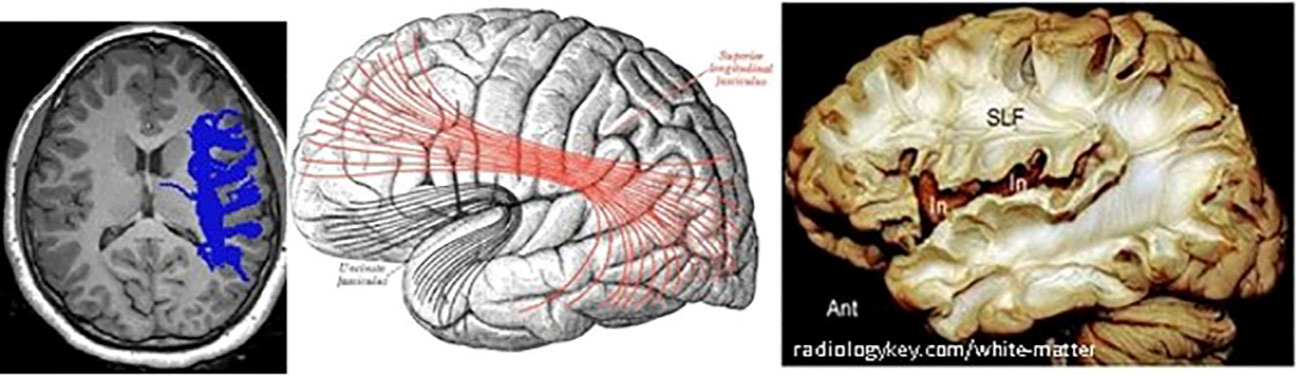
Right SLF white matter. Left: axial view of one diabetes patient (c016), right SLF pathway as traced by AFQ–Center: diagram showing the lateral surface of the left cerebral hemisphere. SLF is at the center, in red. Right: dissection of the human brain from the lateral aspect by the Klingler technique. Resecting the superficial digitations exposes the superior longitudinal fasciculus (SLF) superior to the sylvian fissure and insula (In).

As in other recent research, our results show significant differences in brain matter integrity between healthy adolescents and those with T1D. WM and GM differences were found in cognition-related brain structures, such as the SLF (**Figure 9**) and the corona radiata (CR), and in the GM structures that these tracts connect. Significant group differences were observed in the integrity of several tracts.

Most of our findings concern higher FA, higher AD, or lower RD in the healthy group compared with those with T1D. Most tracts examined were connected to frontal lobe segments or to fibers that connect frontal areas with other lobes, mainly temporal.

Some significant differences were found, however, in the opposite direction. Significantly higher FA and restricted RD in the T1D group compared with healthy subjects were detected in several locations along the posterior parietal segment of the corpus callosum. This may suggest that its fiber bundles are denser in T1D patients than in the healthy group, making it reasonable to hypothesize that brain connectivity in T1D is modified by compensation processes. The posterior parietal segment of the corpus callosum receives fibers from the primary sensory area and connects the somesthetic association cortex with thalamic nuclei. It is mostly referred to as the somesthetic association area that restores our sensation’s memory, permitting assessment, for example, of the characteristics of an object held in the hand (48).

### Associations between brain structure and neurobehavioral functions

VBA group differences were observed between T1D patients and healthy controls in the SLF and the posterior CR. WM connectivity is very important in many spheres—motor, emotional, behavioral, linguistic, and cognitive. These abilities develop through childhood and adolescence and into early adulthood. The SLF connects multiple regions and is involved in language, attention, memory, and executive data processing. Few studies have addressed brain tract structure in adolescents with T1D, and none have focused on its correlation with specific neurocognitive functions (49). Our results concur with others who report decreased WM integrity in several brain areas in T1D (11, 50), in addition to neurocognitive connection to poorer glycemic control (12, 46). Our findings, however, derived using meticulous new methods and specific diabetes-oriented testing, are new in that they demonstrate statistically significant associations between cognitive performance and specific tract properties, WM quality, and neurocognitive performance. Stroop food and nonfood response times are strongly correlated with the integrity of the corpus callosum’s superior frontal segment. It has recently been reported that the prefrontal cortex plays a dominant role in controlling food selection and intake, and its function may change in patients with diabetes due to its dependence on insulin metabolism (51).

Our study has limitations. Its population is smaller than planned because of the difficulty in recruiting adolescents willing to comply with all study procedures, hospitalization, and the lack of gain for the individual involved in the study. This prevented a sufficient number of subjects from demonstrating conclusively significant differences between groups before glycemic parameter considerations. Our study population is, however, a consistent homogeneous demographic group without the confounding variables of age, gender, puberty, comorbidities, quality of life, socioeconomic scale, and T1D complications. A second limitation was the Ethics Committee’s restriction of MRI protocols to 25 min because of the young age of the study population, preventing cognitive tasking with glucose manipulation under imaging (functional MRI).

To conclude, our study sheds new light on cognitive brain domains that may be specifically associated with glycemic control and are strongly linked to missed pre-meal boluses (52, 53). A potentially highly relevant issue, even in this era of hybrid closed-loop therapy, is that the only barrier to not only improving but also controlling the target may be pre-meal bolus delivery.

Our findings emphasize that networks responsible for learning, memory, and controlled reactivity to food may be compromised in adolescents with T1D. We present several exciting results that call for further investigation. Although the associations we discovered comply with the known functions of major brain tracts, further research is needed to identify the processes that establish these associations.

## Data availability statement

The raw data supporting the conclusions of this article will be made available by the authors, without undue reservation.

## Ethics statement

Studies involving human participants were reviewed and approved by Israel’s Health Ministry’s Helsinki Committee. Written informed consent to participate in this study was provided by the participants’ legal guardian and an assent form was signed by the participant.

## Author contributions

EL, RG and MR designed the study, analyzed and interpreted the data, and wrote the manuscript. AL and TS contributed to the study design, collected data, and reviewed and edited the manuscript. ML and EH collected data and reviewed and edited the manuscript. MG, JY, and ES collected data, analyzed and interpreted them, and reviewed and edited the manuscript. MR and EL designed the study, MR and AL were responsible for data collection . EL, RG and MR analyzed and interpreted the results. MR and El are the guarantors of this work. As such, they have full access to the dataset (EL is blinded to the names of the patients) and is responsible for the integrity of the data and the accuracy of the data analysis. All authors contributed to the article and approved the submitted version.

## References

[1] Litmanovitch E Geva R Rachmiel M. Short and long term neuro-behavioral alterations in type 1 diabetes mellitus pediatric population. World J Diabetes (2015) 6:259–70. doi: 10.4239/wjd.v6.i2.259 PMC4360419 25789107

[2] Mauras N Buckingham B White NH Tsalikian E Weinzimer SA Jo B. Impact of type 1 diabetes in the developing brain in children: A longitudinal study. Diabetes Care (2021) 44:983–92. doi: 10.2337/dc20-2125 PMC7985430 33568403

[3] Miles R Root F. Psychologic tests applied to diabetic patients. Arch Intern Med (1922) 30:767–77. doi: 10.1001/archinte.1922.00110120086003

[4] Slattery D Amiel SA Choudhary P. Optimal prandial timing of bolus insulin in diabetes management: A review. Diabetic Med (2018) 35:306–16. doi: 10.1111/dme.13525 PMC5836969 29044708

[5] Tönnies T Imperatore G Hoyer A Saydah SH D’Agostino RB Divers J. Estimating prevalence of type I and type II diabetes using incidence rates: The SEARCH for diabetes in youth study. Ann Epidemiol (2019) 37:37–42. doi: 10.1016/j.annepidem.2019.07.006 31383511 PMC6785183

[6] ADA. 13. children and adolescents: Standards of medical care in diabetes–2019. Diabetes Care (2019) 42:S148–64. doi: 10.2337/dc19-S013 30559239

[7] Prahalad P Zaharieva DP Addala A New C Scheinker D Desai M. Improving clinical outcomes in newly diagnosed pediatric type 1 diabetes: Teamwork, targets, technology, and tight control–the 4T study. Front Endocrinol (2020) 11. doi: 10.3389/fendo.2020.00360 PMC7363838 32733375

[8] Olinder AL Kernell A Smide B. Missed bolus doses: Devastating for metabolic control in CSII-treated adolescents with type 1 diabetes. Pediatr Diabetes (2009) 10:142–8. doi: 10.1111/j.1399-5448.2008.00462.x 19175898

[9] Mauras N Mazaika P Buckingham B Weinzimer S White NH Tsalikian E. Longitudinal assessment of neuroanatomical and cognitive differences in young children with type 1 diabetes: Association with hyperglycemia. Diabetes (2015) 64:1770–9. doi: 10.2337/db14-1445 PMC4407847 25488901

[10] Arbelaez AM Semenkovich K Hershey T. Glycemic extremes in youth with T1DM: The structural and functional integrity of the developing brain. Pediatr Diabetes (2013) 14:541–53. doi: 10.1111/pedi.12088 PMC3857606 24119040

[11] Fox LA Hershey T Mauras N Arbeláez AM Tamborlane WV Buckingham B. Persistence of abnormalities in white matter in children with type 1 diabetes. Diabetologia (2018) 61:1538–47. doi: 10.1007/s00125-018-4610-6 PMC5991628 29654376

[12] DiMeglio LA Kanapka LG DeSalvo DJ Anderson BJ Harrington KR Hilliard ME. Time spent outside of target glucose range for young children with type 1 diabetes: a continuous glucose monitor study. Diabetic Med (2020) 37:1308–15. doi: 10.1111/dme.14276 PMC9065795 32096282

[13] Cato A Hershey T. Cognition and type 1 diabetes in children and adolescents. Diabetes Spectr (2016) 29:197–202. doi: 10.2337/ds16-0036 27899870 PMC5111530

[14] Cameron FJ. The impact of diabetes on brain function in childhood and adolescence. Pediatr Clinics North America (2015) 62:911–27. doi: 10.1016/j.pcl.2015.04.003 26210624

[15] Datye KA Boyle CT Simmons J Moore DJ Jaser SS Sheanon N. Timing of meal insulin and its relation to adherence to therapy in type 1 diabetes. J Diabetes Sci Technol (2017) 12:349–55. doi: 10.1177/1932296817728525 PMC5851213 28895431

[16] van Duinkerken E Snoek FJ de Wit M. The cognitive and psychological effects of living with type 1 diabetes: a narrative review. Diabetic Med (2020) 37:555–63. doi: 10.1111/dme.14216 PMC7154747 31850538

[17] Jongen C Biessels GJ. Structural brain imaging in diabetes: A methodological perspective. Eur J Pharmacol (2008) 585:208–18. doi: 10.1016/j.ejphar.2007.11.085 18407264

[18] Bassett DS Brown JA Deshpande V Carlson JM Grafton ST. Conserved and variable architecture of human white matter connectivity. NeuroImage (2011) 54:1262–79. doi: 10.1016/j.neuroimage.2010.09.006 20850551

[19] Aye T Barnea-Goraly N Ambler C Hoang S Schleifer K Park Y. White matter structural differences in young children with type 1 diabetes: A diffusion tensor imaging study. Diabetes Care (2012) 35:2167–73. doi: 10.2337/dc12-0017 PMC3476914 22966090

[20] Brouwer RM Mandl RC Schnack HG van Soelen IL van Baal GC Peper JS. White matter development in early puberty: A longitudinal volumetric and diffusion tensor imaging twin study. PloS One (2012) 7:e32316. doi: 10.1371/journal.pone.0032316 22514599 PMC3326005

[21] Antenor-Dorsey JAV Meyer E Rutlin J Perantie DC White NH Arbelaez AM. White matter microstructural integrity in youth with type 1 diabetes. Diabetes (2013) 62:581–9. doi: 10.2337/db12-0696 PMC3554385 23139349

[22] Barnea-Goraly N Raman M Mazaika P Marzelli M Hershey T Weinzimer SA. Alterations in white matter structure in young children with type 1 diabetes. Diabetes Care (2013) 37. doi: 10.2337/dc13-1388 PMC3898758 24319123

[23] Biessels GJ Reijmer YD. Brain changes underlying cognitive dysfunction in diabetes: What can we learn from MRI? Diabetes (2014) 63:2244–52. doi: 10.2337/db14-0348 24931032

[24] Mills KL Tamnes CK. Methods and considerations for longitudinal structural brain imaging analysis across development. Dev Cogn Neurosci (2014) 9:172–90. doi: 10.1016/j.dcn.2014.04.004 PMC6989768 24879112

[25] Jeurissen B Descoteaux M Mori S Leemans A. Diffusion MRI fiber tractography of the brain. NMR Biomed (2019) 32:e3785. doi: 10.1002/nbm.3785 28945294

[26] ADA. Standards of medical care in diabetes–2014. Diabetes Care (2014) 37(Suppl. 1):S14–80. doi: 10.2337/dc14-S014 24357209

[27] Miller KM Foster NC Beck RW Bergenstal RM DuBose SN DiMeglio LA. Current state of type 1 diabetes treatment in the U.S.: Updated data from the T1D exchange clinic registry. Diabetes Care (2015) 38:971–8. doi: 10.2337/dc15-0078 25998289

[28] I.C.B.o.S. Characterization and classification of geographical units by the socio-economic level of the population, 2015. (2019).

[29] Goldstein A Haelyon U Krolik E Sack J. Comparison of body weight and height of Israeli schoolchildren with the tanner and centers for disease control and prevention growth charts. Pediatrics (2001) 108 (6):E108. doi: 10.1542/peds.108.6.e108 11731635

[30] Marshall WA Tanner JM. Growth and physiological development during adolescence. Annu Rev Med (1978) 19:283–300. doi: 10.1146/annurev.me.19.020168.001435 4297619

[31] Reynolds CR Bigler ED. Factor structure, factor indexes, and other useful statistics for interpretation of the test of memory and learning (TOMAL). Arch Clin Neuropsychol (1996) 11:29–43. doi: 10.1093/arclin/11.1.29

[32] Thaler NS. Cluster analysis of the TOMAL standardization sample. Developmental Psychology, University of Nevada, Las Vegas (2010).

[33] Gerstadt CL Hong YJ Diamond A. The relationship between cognition and action: performance of children 3 1/2-7 years old on a stroop-like day-night test. Cognition (1994) 53:129–53. doi: 10.1016/0010-0277(94)90068-X 7805351

[34] Ramon D Geva R Goldstein A. Trait and state negative affect interactions moderate inhibitory control performance in emotionally loaded conditions. Pers Individ Dif (2011) 51:95–101. doi: 10.1016/j.paid.2011.03.016

[35] Miyake A Friedman NP Emerson MJ Witzki AH Howerter A Wager TD. The unity and diversity of executive functions and their contributions to complex “Frontal lobe” tasks: A latent variable analysis. Cogn Psychol (2000) 41:49–100. doi: 10.1006/cogp.1999.0734 10945922

[36] Kraut M Calhoun V Pitcock J Cusick C Hart J. Neural hybrid model of semantic object memory: Implications from event-related timing using fMRI. J Int Neuropsychol Soc: JINS (2003) 9:1031–40. doi: 10.1017/S135561770397007X 14738284

[37] Assaf M Calhoun VD Kuzu CH Kraut MA Rivkin PR Hart J. Neural correlates of the object-recall process in semantic memory. Psychiatry Res: Neuroimaging (2006) 147:115–26. doi: 10.1016/j.pscychresns.2006.01.002 16938439

[38] Yeatman JD Dougherty RF Myall NJ Wandell BA Feldman HM. Tract profiles of white matter properties: Automating fiber-tract quantification. PloS One (2012) 7:e49790. doi: 10.1371/journal.pone.0049790 23166771 PMC3498174

[39] Mori S van Zijl PCM. Fiber tracking: priciples and strategies - a technical review. NMR Biomed (2002) 15:468–80. doi: 10.1002/nbm.781 12489096

[40] Huang H Zhang J Jiang H Wakana S Poetscher L Miller MI. DTI tractography based parcellation of white matter: Application to the mid-sagittal morphology of corpus callosum. NeuroImage (2005) 26:195–205. doi: 10.1016/j.neuroimage.2005.01.019 15862219

[41] Dougherty RF Ben-Shachar M Deutsch GK Hernandez A Fox GR Wandell BA. Temporal-callosal pathway diffusivity predicts phonological skills in children. Proc Natl Acad Sci U.S.A. (2007) 104:8556–61. doi: 10.1073/pnas.0608961104 PMC1895988 17483487

[42] Mori S Crain B Chacko VP van zijl P. Three dimensional tracking of axonal projections in the brain by magnetic resonance imaging. annal neurol 45: 265-269. Ann Neurol (1999) 45:265–9. doi: 10.1002/1531-8249(199902)45:2<265::AID-ANA21>3.0.CO;2-3 9989633

[43] Sasson E Doniger GM Pasternak O Tarrasch R Assaf Y. White matter correlates of cognitive domains in normal aging with diffusion tensor imaging. Front Neurosci (2013) 7:32–2. doi: 10.3389/fnins.2013.00032 PMC3595518 23493587

[44] Nichols TE Holmes AP. Nonparametric permutation tests for functional neuroimaging: A primer with examples. Hum Brain Mapp (2002) 15:1–25. doi: 10.1002/hbm.1058 11747097 PMC6871862

[45] Ryan CM. Why is cognitive dysfunction associated with the development of diabetes early in life? diathesis hypothesis. Pediatr Diabetes (2006) 7:289–97. doi: 10.1111/j.1399-5448.2006.00206.x 17054452

[46] van Duinkerken E Ryan CM. Diabetes mellitus in the young and the old: Effects on cognitive functioning across the life span. Neurobiol Dis (2020) 134:104608. doi: 10.1016/j.nbd.2019.104608 31494283

[47] Aye T Reiss AL Kesler S Hoang S Drobny J Park Y. The feasibility of detecting neuropsychologic and neuroanatomic effects of type 1 diabetes in young children. Diabetes Care (2011) 34:1458–62. doi: 10.2337/dc10-2164 PMC3120162 21562318

[48] Kesner RP. The posterior parietal cortex and long-term memory representation of spatial information. Neurobiol Learn Memory (2009) 91:197–206. doi: 10.1016/j.nlm.2008.09.004 PMC2675271 18835456

[49] Pourabbasi A Tehrani-Doost M Qavam SE Arzaghi SM Larijani B. Association of diabetes mellitus and structural changes in the central nervous system in children and adolescents: A systematic review. J Diabetes Metab Disord (2017) 16:10. doi: 10.1186/s40200-017-0292-8 28271054 PMC5335845

[50] Toprak H Yetis H Alkan A Filiz M Kurtcan S Aralasmak A. Relationships of DTI findings with neurocognitive dysfunction in children with type 1 diabetes mellitus. Br J Radiol (2016) 89:20150680. doi: 10.1259/bjr.20150680 26728951 PMC4986488

[51] Kullmann S Kleinridders A Small DM Fritsche A Häring H-U Preissl H. Central nervous pathways of insulin action in the control of metabolism and food intake. Lancet Diabetes Endocrinol (2020) 8:524–34. doi: 10.1016/S2213-8587(20)30113-3 32445739

[52] Gomes MB Negrato CA. Adherence to insulin therapeutic regimens in patients with type 1 diabetes. a nationwide survey in Brazil. Diabetes Res Clin Pract (2016) 120:47–55. doi: 10.1016/j.diabres.2016.07.011 27513598

[53] Bermeo-Cabrera J Almeda-Valdes P Riofrios-Palacios J Aguilar-Salinas CA Mehta R. Insulin adherence in type 2 diabetes in Mexico: Behaviors and barriers. J Diabetes Res (2018) 2018:3190849. doi: 10.1155/2018/3190849 30116737 PMC6079463

